# Editorial: Psychiatric Comorbidities in Children and Adolescents With ASD and in Typically Developing Children

**DOI:** 10.3389/fpsyt.2021.817978

**Published:** 2022-01-03

**Authors:** Marija Raleva, Vaska Stancheva-Popkostadinova, Milica Pejovic-Milovancevic

**Affiliations:** ^1^Faculty of Medicine, Ss. Cyril and Methodius University, Skopje, North Macedonia; ^2^Department of Medical Social Sciences, South-West University “Neofit Rilski”, Blagoevgrad, Bulgaria; ^3^Faculty of Medicine, University of Belgrade, Belgrade, Serbia

**Keywords:** atypical development, typical development, autism spectrum disorder, comorbidity, adverse childhood experiences

The conceptualization of Autistic Spectrum Disorder (ASD) has changed from a condition that is narrowly defined and rare, to one that is defined by a wider set of diagnostic criteria and is far more common in the population. The number of people diagnosed with autism has jumped by 787% between 1998 and 2018 (a percentage increase from 100% modeled at baseline year in 1998), which is likely an effect of increasing recognition (1). The overall ASD prevalence is 23.0 per 1,000 (one in 44) children aged 8 years (2).

Atypical development as part of ASD has been examined by numerous researchers and usually compared to typical development of children and adolescents. A typically developing child acquires specific skills and behaviors according to a predictable rate and sequence, i.e., developmental milestones, whereas atypically developing children miss or fail to meet anticipated milestones. Although developmental trajectories differ from one group to another, the neurodiversity perspective in autism research, acknowledges that autistic traits represent an individual difference that, in the appropriate context, may enhance (or at least not impair) a person's social, occupational, or academic functioning (3, 4).

However, there is also a growing recognition that ASD is a lifelong condition that co-occurs with other disorders. The so called “co-occurring conditions” or comorbidities can appear during childhood or later in adolescence or adulthood. Diagnosis of comorbidities can be challenging because many people with ASD have difficulties recognizing and communicating their symptoms.

Comorbidities in children with ASD often have a significant impact on their functioning. They can exacerbate existing autism symptoms and make treatment less successful, and can give rise to or make worse other comorbidities in an individual. An extensive research body on the impact of environmental or genetic factors on comorbidities (motor, emotional, cognitive, and social) in children with typical development has been performed. There is a significant lack of such research in children with ASD. Those factors have developmental impact on both typical as well as on atypical development and can contribute to comorbidities in both groups of children across the life span.

A number of the papers in the Research Topic focus on questions with which the field has been grappling for decades: comorbidity in relation to co-occurring neuropsychiatric disorders in infants, children, adolescents, and young adults across the lifespan.

In her brief research report, Ivanović presented a cross-sectional study on the prevalence of psychiatric comorbidities in population of children with ASD in one referral center—Autism Center in Montenegro. The context they work in is challenging one because there are only two child psychiatrists who cover more than 620,000 inhabitants. The research communicated not only heterogeneity but also multiple presence of psychiatric comorbidities.

Tololeski et al. reviewed the state of research on high-functioning ASD and anorexia nervosa (AN) comorbidity in children and adolescents. They emphasize the need for more effective and holistic treatment of this comorbidity. Cognitive remediation therapy and the promising pharmaco-therapeutic candidate oxytocin, were highlighted as first guidelines to treat this comorbidity.

Presenting a case study of ASD patient with gender dysphoria comorbidity, identified and treated during 3.5 years, Zupanič et al. presented the main problems with diagnosis and treatment, and stress the advantage of broad team approach and broad therapeutic interventions for improvement the patient's symptoms.

Milovanovic and Grujicic in their review explored epilepsy and ASD comorbidity, highlighting the correlation of the underlined conditions that can contribute to the altered neurophysiology of the brain circuits. They also suggest that epileptic activity can lead to cognitive and behavioral impairment beyond the underlying pathology. The application of the electroencephalography (EEG) as a diagnostic method can further our understanding of ASD neurophysiology, and should be part of the clinical investigation of children with ASD.

In line with these papers on comorbidity in ASD, Manolova et al. present an original toolkit for detailed assessment of psychological functioning of children with ASD in the early life years. Based on their 20 years of experience, they found it important both for delineating Individual profile characteristics, as well as for the differential diagnosis and the detection of comorbid conditions.

Two papers in the Research Topic focus on questions on genetic mutations and polymorphisms in ASD research, which may contribute to the underlying neurodevelopmental basis of behavioral and cognitive impairment in children with typical and atypical development. Steele et al. in their paper recognized that germline heterozygous PTEN mutations are associated with high prevalence of ASD and elevated rates and severity of behavioral problems. By comparing patients with PTEN mutation and ASD and PTEN mutations without an ASD diagnosis to ASD patients with macrocephaly that lack PTEN mutations this paper sheds insight into subtle clinical differences specific to each group. Having quantitative data characterizing PTEN-ASD patients is an important step in being able to identify the ASD endo-phenotype of this genetically identifiable subpopulation of ASD.

Mandic-Maravic et al. found that glutathione S-transferase polymorphisms (GST) are related to cognitive and adaptive abilities in children with ASD, influencing the severity of symptoms, and have shown that specific genotypes have a predictive value on the level of non-verbal communication impairment and on chances of having seizures during life, while others, such as GSTM1-active genotype predicted higher adaptive functioning and GSTP1^*^ allele genotype was significantly associated to better cognitive functioning in children with ASD. The study combines both genetic and environmental factors and their effect on clinical and intellectual/adaptive characteristics in ASD patients.

In the last paper, Uršič et al. made an attempt to validate Lifetime Incidence of Traumatic Events Questionnaires (LITE-S/P), an instrument for screening and assessing traumatic events in children with typical development. Their aim was to provide a short checklist for screening and assessing the traumatic events in the child's life, due to the lack of such instruments in research and clinical practice in Slovenia.

In summary, the papers in the Research Topic illustrate how much we have advanced in understanding typical and atypical development in relation to comorbidities than before. They also remind us of how much farther we must go to realize the purpose of our field to prevent disorder, treat it effectively, and understand the causes and consequences of typical and atypical development in all its heterogeneity. The authors who have contributed to the Research Topic highlight the current state of our science and practice, but they also give rise to exiting new directions for research.

## Author Contributions

MR, VS-P, and MP-M edited the manuscripts in the Research Topic and contributed to this editorial. All three authors critically reviewed and provided the feedback on the initial versions of these manuscripts and approved the final version of the manuscripts. All authors contributed to the article and approved the submitted version.

## Conflict of Interest

The authors declare that the research was conducted in the absence of any commercial or financial relationships that could be construed as a potential conflict of interest.

## Publisher's Note

All claims expressed in this article are solely those of the authors and do not necessarily represent those of their affiliated organizations, or those of the publisher, the editors and the reviewers. Any product that may be evaluated in this article, or claim that may be made by its manufacturer, is not guaranteed or endorsed by the publisher.
